# Bibliographical

**Published:** 1893-06

**Authors:** 


### Bibliographical.

A Compend of Dental Pathology and Dental Medicine, containing the most noteworthy points on the subjects of interest to the Dental Student, and a section on Emergencies, by George W. Warren, D.D.S., Chief of the Clinical Staff, Pennsylvania College of Dental Surgery, Philadelphia. Second Edition, illustrated.

This little work presents brief but very concise statements in regard to the development of the teeth-structure, anatomy, pathology, and injuries. While the descriptions are very plain and concise they will fail to satisfy the student, but, what is far better they lead him to a point where he desires to know more upon the subject, and really, in a measure prepare him for what is presented in the more elaborate works.

The second part of the work is devoted to dental medicine, and the same may be said of it as of the first—that it presents the topics embraced in a very brief but clear and concise form, and a mastering of these will prepare the student for more profitably studying the larger works. It should be in the hands of every dental student, and of every practitioner as well.

The third part is an appendix, giving the methods of procedure in emergencies. Its suggestions in many particulars, at least, are valuable not only for the physician and dentist, but for the laity as well, and on that account the work would be valuable in every household.

The work is published by P. Blakiston, Son & Co., Philadelphia, and is for sale by Robt. Clarke, Cincinnati, and doubtless can be obtained through booksellers generally.

